# Normalization strategies differently affect circulating miRNA profile associated with the training status

**DOI:** 10.1038/s41598-019-38505-x

**Published:** 2019-02-07

**Authors:** Martina Faraldi, Marta Gomarasca, Veronica Sansoni, Silvia Perego, Giuseppe Banfi, Giovanni Lombardi

**Affiliations:** 1 Laboratory of Experimental Biochemistry & Molecular Biology, IRCCS Istituto Ortopedico Galeazzi, Milano, Italy; 2 Vita-Salute San Raffaele University, Milano, Italy; 3 Gdańsk University of Physical Education & Sport, Gdańsk, Poland

## Abstract

MicroRNAs are fine regulators of the whole-body adaptive response but their use as biomarkers is limited by the lack of standardized pre- and post-analytical procedures. This work aimed to compare different normalization approaches for RT-qPCR data analyses, in order to identify the most reliable and reproducible method to analyze circulating miRNA expression profiles in sedentary and highly-trained subjects. As the physically active status is known to affect miRNA expression, they could be effective biomarkers of the homeostatic response. Following RNA extraction from plasma, a panel of 179 miRNAs was assayed by RT-qPCR and quantified by applying different normalization strategies based on endogenous miRNAs and exogenous oligonucleotides. hsa-miR-320d was found as the most appropriate reference miRNA in reducing the technical variability among the experimental replicates and, hence, in highlighting the inter-cohorts differences. Our data showed an association between the physically active status and specific skeletal muscle- and bone-associated circulating miRNAs profiles, revealing that established epigenetic modifications affect the baseline physiological status of these tissues. Since different normalization strategies led to different outputs, in order to avoid misleading interpretation of data, we remark the importance of the accurate choice of the most reliable normalization method in every experimental setting.

## Introduction

MicroRNAs (miRNAs) are endogenous non-coding RNAs involved in the post-transcriptional regulation of gene expression by inducing the degradation of the target mRNA and/or by inhibiting its translation into a protein, as reviewed in^1^. miRNAs are expressed by all cell types and tissues and, in response to physiological stimuli and pathological processes, they can be released into the circulation^2^. The discovery of circulating miRNAs have opened the possibility of utilizing their quantification in biological fluids for monitoring specific biological processes. Indeed, circulating miRNAs, and particularly their free form, are currently under investigation as potential biomarkers of several diseases, such as cancers, cardiovascular diseases, skeletal muscle and bone diseases, and inflammatory conditions^3^.

However, several challenges must be considered when circulating miRNAs are used as biomarkers. Since miRNAs are often present at very low concentrations in serum and plasma, their quantification requires highly sensitive and specific methods as reverse transcription quantitative real time polymerase chain reaction (RT-qPCR)^4^. Nevertheless, some technical variables introduced during the experimental steps, e.g., starting sample amount, collection modality, storage condition, and miRNA isolation/transcription efficiency, profoundly affect the final amount of miRNAs, eventually masking the true biological response. Importantly, the normalization method used, i.e., the mathematical process ranking the set of candidate genes according to their expression stability in a given sample set and in a given experimental design, strongly influences the analytical output of the RT-qPCR. The purpose of normalization is to reduce the analytical variability in order to obtain the most reliable and reproducible biological result. Therefore, the choice of the appropriate normalizer(s) is a crucial aspect in miRNA quantification, especially for their implementation in a clinical setting. Among the normalization strategies used in RT-qPCR analysis of circulating miRNAs, the most common are based on exogenous synthetic oligonucleotides, geometrical mean of the Cq (quantification cycle) of the whole set of miRNA analyzed, and endogenous miRNAs^5^. Several studies have used exogenous synthetic oligonucleotides as reference genes. Exogenous oligonucleotides (such as cel-miR-39, cel-miR-54 or cel-miR-238 from *Caenorhabditis elegans*)^6,7^ are added at known concentration in the biological samples in order to monitor processes related to miRNA analysis (e.g., RNA extraction and reverse-transcription). Therefore, they only correct qPCR data for the variability related to these specific processes but not for the other previously described intrinsic variables to which they are not exposed, contrarily to endogenous miRNAs. Endogenous miRNAs might be considered as optimal reference genes, since their expression is affected by the same variables as the target genes. Currently, a universally accepted normalization strategy based on endogenous miRNAs is still lacking and the main strategies are based on either a single reference miRNA or a combination (mean) of more miRNAs. This latter is considered as the approach that drastically reduces the effect of intra- and inter-kinetic variability in RT-qPCR (sample-to-sample and run-to-run variations) compared to the single gene-based normalization^8^. Another endogenous miRNAs-based normalization strategy uses the averaged Cq value of all the analyzed miRNAs (global mean)^9^, a strategy commonly used in experiments assaying large numbers of miRNAs and/or in experiments in which stable reference genes are unknown. According to these different methodological approaches, many algorithms, such as geNorm^8^, NormFinder^10^ and BestKeeper^11^, have been developed to identify the most stable endogenous genes to use as reference, under a specific experimental condition. Currently, there are no definitive guidelines ruling data normalization in miRNA expression analysis although it is clear that different normalization strategies give rise to very different results, with the (too high) risk of generating confusion^12^.

Physical activity (PA) triggers the adaptive response that keeps the whole-body homeostasis during exercise stimulation. This adaptive response relies on the alteration of gene expression and allows the regulation of several physiological processes including myocardial and skeletal muscle metabolism, regeneration and remodeling, mitochondrial biogenesis, inflammation, and angiogenesis. The epigenetic regulation, as that carried on by miRNAs, is of fundamental importance in determining the physiological outcomes of the adaptation^13^. Several studies have demonstrated the beneficial effects of PA in preventing or delaying the progression of a number of pathological conditions^14^. Changes in circulating miRNA levels in response to exercise has been investigated, although only partially, in order to establish the role of these molecules as mediators of the physiological processes associated with exercise^13,15^. Based on this background, this work was designed to study the post-analytical variability associated with the normalization strategy adopted in data analysis that can ultimately affect the biological outcome. Since PA has profound effects on the pan-miRNAs expression profile and this can in turn affect the diagnostic value of a miRNA, we chose, as study cohorts, sedentary healthy subjects and their highly-trained counterpart. The differences in miRNA profile between the two cohorts were studied by applying four normalization strategies based on: exogenous oligonucleotides, global mean, mean of the most stable miRNAs ranked by NormFinder, and single most stable endogenous miRNAs.

## Results

### Selection of suitable endogenous miRNAs as reference genes

**Figure 1:**
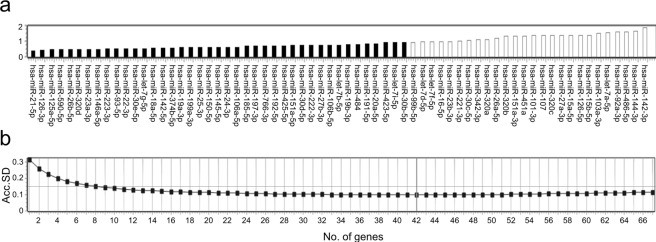
Analyses on endogenous miRNAs. (**a**) Arbitrary stability value of 67 miRNAs selected by NormFinder. miRNAs are ranked as stable (black) and unstable (white). (**b**) Acc.SD of 67 miRNAs selected by NormFinder. The lowest Acc.SD. value indicates the optimal number of reference genes. Expression stability analysis was performed comparing free-circulating miRNAs from sedentary and trained subjects.

To define the set of endogenous miRNAs suitable as reference genes among the 179 analyzed, the stability of expressed miRNAs was evaluated by the NormFinder algorithm. The algorithm calculated the stability of only those miRNAs expressed in all replicates of both sedentary and trained groups and the final output brought to the selection of 67 miRNAs. Figure 1a shows the identified endogenous reference miRNAs ranked according to their arbitrary stability value. Among the 67 miRNAs, according to the accumulated standard deviation (Acc.SD), NormFinder identified 41 miRNAs as the most stable ones (Figure 1b). To avoid the presence of miRNAs co-regulated by the same transcriptional and epigenetic mechanisms, only the most stable miRNA of each cluster was included in the calculation of the averaged value. miRBase (http://www.mirbase.org/) was used to find miRNAs belonging to the same cluster (distance <10 kb). This led to the final inclusion of 33 reference miRNAs (Table S1).

### Comparison of normalization strategies based on endogenous miRNAs

**Figure 2:**
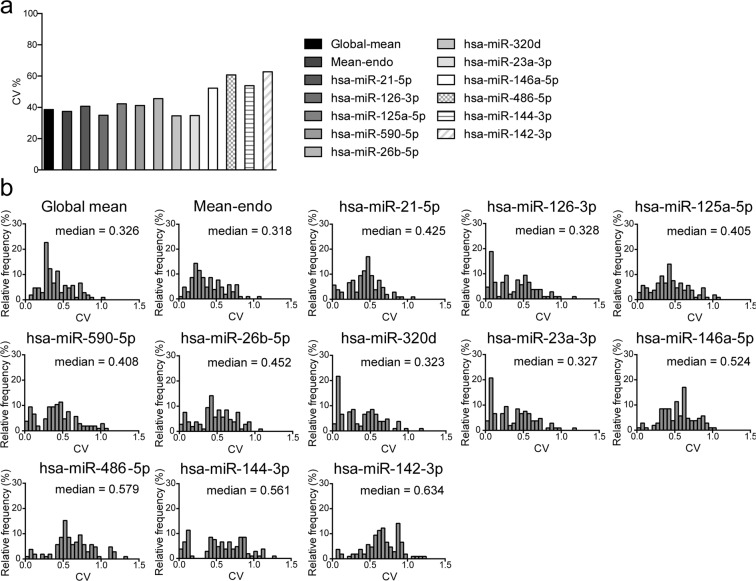
Coefficient of variation analyses after normalization on endogenous miRNAs. (**a**) Coefficient of variation (CV%) of all analyzed miRNAs normalized on global mean, mean-endo, 8 most stable miRNAs, singularly (hsa-miR-21-5p, hsa-miR-126-3p, hsa-miR-125a-5p, hsa-miR-590-5p, hsa-miR-26b-5p, hsa-miR-320d, hsa-miR-23a-3p, and hsa-miR-146a-5p) and 3 less stable miRNAs, singularly (hsa-miR-486, hsa-miR-144-3p, hsa-miR-142-3p). The lowest CV% indicates the most suitable method. (**b**) Frequency distribution of CV% of all analyzed miRNAs normalized as in (**a**). The median CV% distribution is indicated for each graph.

The main purpose of RT-qPCR data normalization is limiting the technical variability. The coefficient of variation (CV) was assessed to evaluate the inter-assay variability among the RT-qPCR replicates. The CV was calculated as the ratio between standard deviation and mean of each miRNA replicates averaged for each group of subjects after normalization: the lower the CV is, the more reliable and reproducible the normalization method is^9,16^. CV of data obtained after normalization on global mean, mean of the Cq of the 33 selected endogenous reference miRNAs (mean-endo), Cq of each of the first 8 most stable miRNAs (hsa-miR-21-5p, hsa-miR-126-3p, hsa-miR-125a-5p, hsa-miR-590-5p, hsa-miR-26b-5p, hsa-miR-320d, hsa-miR-23a-3p, and hsa-miR-146a-5p), and Cq of each of the last 3 less stable miRNAs (hsa-miR-486-5p, hsa-miR-144-3p, hsa-miR-142-3p) identified by NormFinder, were compared. The 8 most stable miRNAs were selected considering an Acc.SD threshold of 50%. As a term of comparison, also the last 3 less stable miRNAs ranked by NormFinder were used as normalizers. Figure 2a shows that data normalized on mean-endo and global mean had CV values of 37.44% and 38.62%, respectively. When considering data normalized on single miRNAs, normalization on the sixth most stable ranked miRNA (hsa-miR-320d) had the lowest CV value (34.62%), while normalizing on the less stable miRNAs gave rise to the highest CV values (ranging from 53.89% to 62.83%). The percentage of the frequency distributions of the CV of all miRNAs, based on the different normalization strategies, is shown in Figure 2b. The percentage of the frequency distribution of CV following normalization on mean-endo and global mean were narrower than those obtained with the other normalization methods. Additionally, the median value of the frequencies distribution of data normalized on mean-endo was comparably lower than that obtained normalizing on hsa-miR-320d alone. Normalization on the less stable miRNAs gave rise to the highest median values. These results support the use of mean-endo or hsa-miR-320d to improve normalization in this specific setting.

### Comparison of normalization strategies based on exogenous oligonucleotides

**Figure 3:**
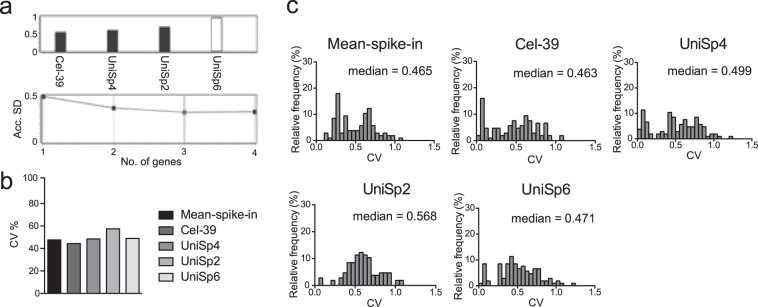
Analyses on exogenous oligonucleotide. (**a**) Arbitrary stability value and Acc.SD. of exogenous oligonucleotide (cel-39, UniSp2, UniSp4 and UniSp6) calculated by NormFinder. Spike-ins are ranked as stable (black) and unstable (white). The analysis of the expression stability was performed comparing free-circulating miRNAs from sedentary and trained subjects. (**b**) CV% of all analyzed miRNAs normalized on mean-spike-in (average of cel-39, UniSp2 and UniSp4) and on cel-39, UniSp2, UniSp4 and UniSp6, singularly. (**c**) Percentage of the frequency distribution of CV of all analyzed miRNAs normalized as in (**b**). Median of the CV% distribution is indicated for each graph.

UniSp2, UniSp4, UniSp5, UniSp6 and cel-miR-39 (cel-39) (i.e., spike-in controls) were added at known amounts in each biological sample in order to estimate the efficiency of RNA extraction and reverse transcription (RT) reaction. In order to evaluate their potential use as reference genes, spike-ins stability was calculated using NormFinder algorithm. UniSp5 was excluded from the analyses due to its low Cq values. Figure 3a shows the spike-ins ranked by arbitrary stability values and the related Acc.SD. NormFinder identified the averaged Cq value of 3 spike-ins, cel-39, UniSp2, and UniSp4 (mean-spike-in), as the most suitable exogenous genes-based normalization method in this sub-analysis. Figure 3b shows the CV of miRNAs expression normalized on mean-spike-in and on cel-39, UniSp2, UniSp4, or UniSp6, singularly. These results indicate that data normalized on mean-spike-in had an averaged CV (47.55%) comparable to those obtained after normalization on cel-39, UniSp4, and UniSp6 (44.40%, 48.50%, and 48.88%, respectively). The analysis of the percentage of the frequency distribution of CV (Fig. 3c) shows that data normalized on mean-spike-in had a median value lower than those normalized on the single spike-ins.

### Effect of different normalization methods on miRNA relative expression profiles

Different normalization methods may affect the result of the expression levels of circulating miRNAs. To evaluate the differences in miRNA expression profiles between sedentary and trained subjects, RT-qPCR data were normalized on global mean and mean-endo, generally considered the most appropriate normalization methods in case of large datasets^9^, hsa-miR-21a-5p, the most stable miRNA ranked by NormFinder, and hsa-miR-320d, the miRNA showing the lowest CV value. Normalization on mean-spike-in and cel-39 were chosen among the exogenous based normalization methods as terms of comparison. The number of miRNAs up- or down-regulated ≥±5-fold in plasma of trained subjects varied according to the normalization method applied, as shown in Table 1.

**Table 1:** Normalization methods and relative number of ≥±5-fold up- or down-regulated miRNAs in trained subject compared to sedentary subjects.

Normalization methods	miRNAs up-regulated	miRNAs down-regulated
Global mean	43	11
Mean-endo	43	13
hsa-miR-21a-5p	41	15
hsa-miR-320d	40	17
Mean-spike	55	8
cel-39	74	9

**Figure 4:**
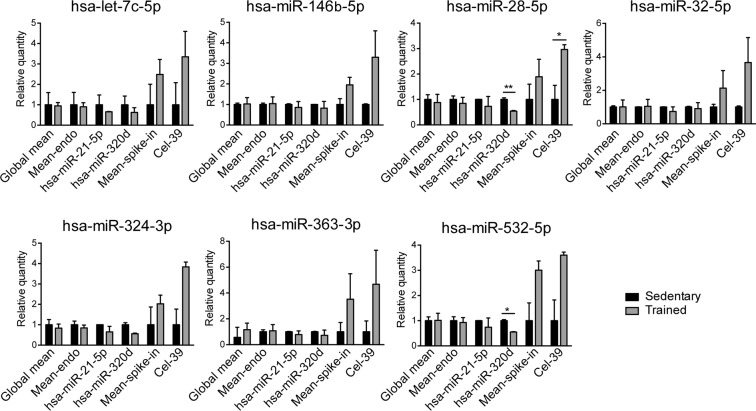
Expression profile of miRNAs of sedentary and trained subjects after normalization on global mean, mean-endo, hsa-miR-21a-5p, hsa-miR-320d, mean-spike-in and cel-39. Expression profile of representative miRNAs selected as >20% up- or down-regulated in plasma of trained subject compared to sedentary subjects (hsa-let-7c-5p, hsa-miR-28-5p, hsa-miR-32-5p, hsa-miR-146b-5p, hsa-miR-324-3p, hsa-miR-363-3p, and miR-532-5p) Statistical analysis was performed with Prism® v6.01 (GraphPad Software). All data are expressed as mean ± SEM and data were compared throughout unpaired *t*-test with Welch’s correction. The differences were considered significant when p value < 0.05 (*p < 0.05, **p < 0.01).

To further investigate the effect of the normalization strategy on miRNA expression levels and on reduction of the technical error among the experimental replicates, the expression profile of circulating miRNAs was analyzed (Fig. 4). This analysis was performed on those miRNAs that were >20% up- or down-regulated in trained subjects compared to sedentary when normalized on global mean, which is considered the best normalization method to analyze a large number of miRNAs: hsa-let-7c-5p, hsa-miR-28-5p, hsa-miR-32-5p, hsa-miR-146b-5p, hsa-miR-324-3p, hsa-miR-363-3p, and hsa-miR-532-5p (Fig. 4).

These results showed that normalization on global mean, mean-endo, hsa-miR-21-5p, and hsa-miR-320d gave similar expression profiles for each miRNA. hsa-miR-28-5p and hsa-miR-532-5p were significantly different between sedentary and trained subjects only when normalized on hsa-miR-320d. This suggests that normalization on hsa-miR-320d is more sensitive than other strategies in highlighting differences in plasma miRNAs of our cohorts. Finally, data normalized on mean-spike-in and cel-39 showed marked differences in the fold-change expression levels, compared to the other normalization methods, but with a completely inverted trend.

### Plasma miRNA expression levels in trained vs sedentary subjects

To study the adaptive response to high-level physical activity (habitual sky runners), plasma miRNA expression profile was compared between sedentary and trained subjects, using hsa-miR-320d as the best fitting normalizer. Table 2 shows the expression levels of miRNAs which are ≥±5-fold up-regulated or down-regulated in highly-trained group compared to sedentary group.

**Table 2:** List of the miRNAs ≥±5-fold up or downregulated in trained subjects compared to sedentary subjects and relative fold-change.

miRNAs up-regulated in trained vs. sedentary		miRNAs down-regulated in trained vs. sedentary	
miRNA	fold-change	miRNA	fold-change
hsa-miR-1	32.15	hsa-let-7a-5p	6.79
hsa-miR-1260a	65.65	hsa-miR-101-3p	5.08
hsa-miR-127-3p	33.73	hsa-miR-103a-3p	8.83
hsa-miR-130a-3p	44.82	hsa-miR-107	8.23
hsa-miR-130b-3p	46.32	hsa-miR-122-5p	9.64
hsa-miR-132-3p	83.51	hsa-miR-125b-5p	96.84
hsa-miR-144-5p	47.87	hsa-miR-140-5p	61.41
hsa-miR-148b-3p	32.66	hsa-miR-142-3p	12.32
hsa-miR-155-5p	32.98	hsa-miR-144-3p	12.94
hsa-miR-16-2-3p	34.06	hsa-miR-181a-5p	65.30
hsa-miR-186-5p	33.45	hsa-miR-199a-5p	132.29
hsa-miR-200c-3p	53.41	hsa-miR-29a-3p	65.45
hsa-miR-20b-5p	33.93	hsa-miR-301a-3p	5.44
hsa-miR-2110	46.39	hsa-miR-30c-5p	5.32
hsa-miR-28-3p	33.37	hsa-miR-331-3p	95.86
hsa-miR-29b-3p	34.48	hsa-miR-378a-3p	32.68
hsa-miR-29c-3p	66.13	hsa-miR-424-5p	32.77
hsa-miR-30e-3p	32.98		
hsa-miR-324-5p	52.15		
hsa-miR-326	34.71		
hsa-miR-335-3p	33.39		
hsa-miR-361-5p	79.73		
hsa-miR-374a-5p	42.27		
hsa-miR-376a-3p	32.73		
hsa-miR-376c-3p	44.99		
hsa-miR-382-5p	33.17		
hsa-miR-409-3p	35.54		
hsa-miR-421	32.35		
hsa-miR-423-3p	33.45		
hsa-miR-425-3p	45.36		
hsa-miR-454-3p	45.44		
hsa-miR-495-3p	32.96		
hsa-miR-502-3p	32.98		
hsa-miR-505-3p	33.85		
hsa-miR-584-5p	51.92		
hsa-miR-629-5p	34.61		
hsa-miR-660-5p	51.57		
hsa-miR-874-3p	32.95		
hsa-miR-877-5p	46.34		
hsa-miR-93-3p	32.87		

It is evident from these results that the physically active status of the highly-trained group is associated with a defined different circulating miRNA profile, compared to the sedentary group. Indeed, among the 179 analyzed circulating miRNAs, 40 were found up-regulated while 17 were down-regulated.

**Figure 5:**
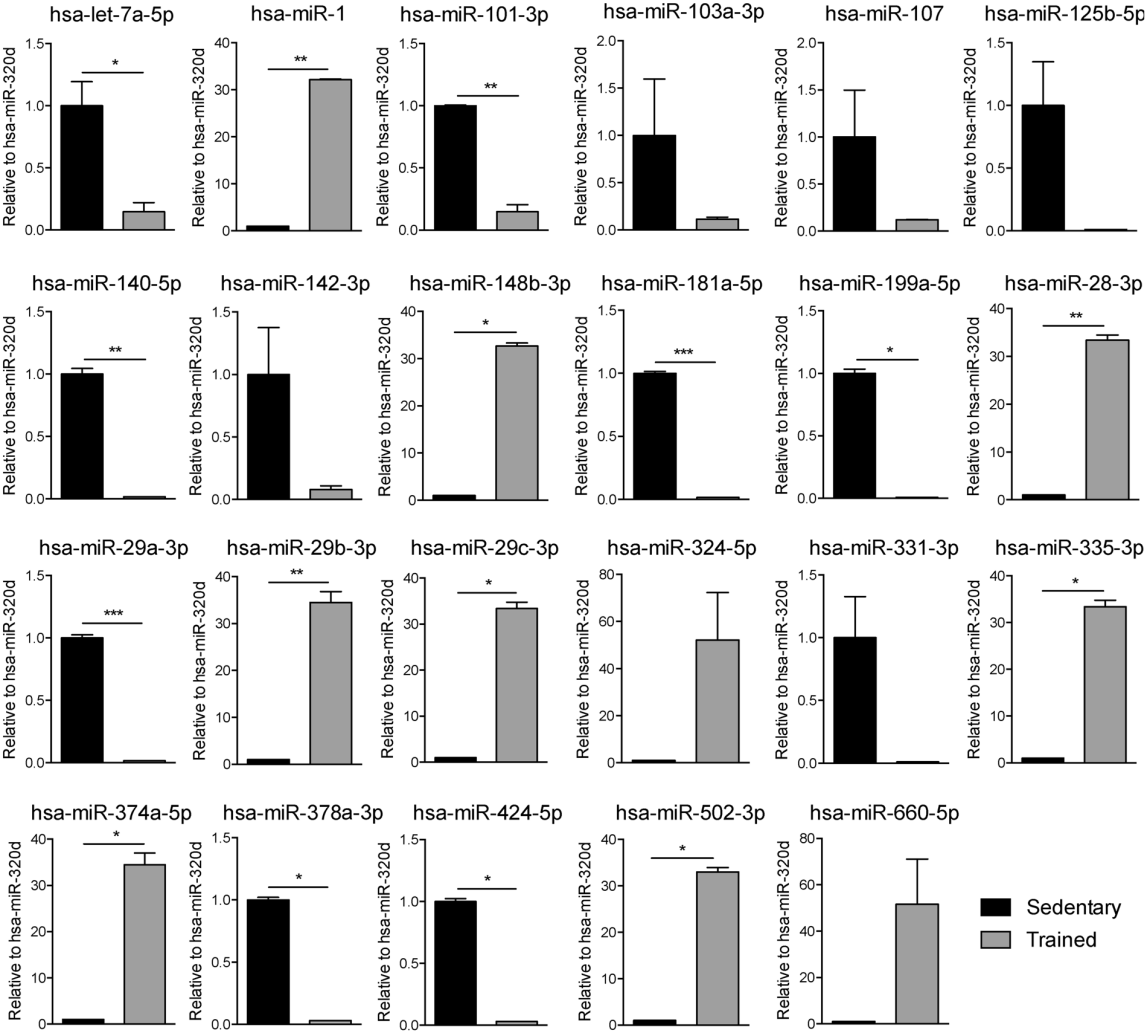
Expression profile of miRNAs of sedentary and trained subjects associated to skeletal muscle and bone. Expression profile of miRNAs from sedentary and trained subjects which function is associated to skeletal muscle and bone (hsa-let-7a-5p, hsa-miR-1, hsa-miR-101-3p, hsa-miR-103a-3p, hsa-miR-107, hsa-miR-125b-5p, hsa-miR-140-5p, hsa-miR-142-3p, hsa-miR-148b-5p, hsa-miR-181a-5p, hsa-miR-199a-5p, hsa-miR-28-3p, hsa-miR-29a-3p, and hsa-miR-29b/c-3p, hsa-miR-324-5p, hsa-miR-331-3p, hsa-miR-335-5p, hsa-miR-374a-5p, hsa-miR-378-3p, miR-424-5p, hsa-miR-502-3p, and hsa-miR-660-5p). Statistical analysis was performed with Prism® v6.01 (GraphPad Software). All data are expressed as mean ± SEM and data were compared throughout unpaired *t*-test with Welch’s correction. The differences were considered significant when p value < 0.05 (*p < 0.05, **p < 0.01, ***p < 0.001).

Based on their expression and function in human skeletal muscle and bone in both physiological and pathological conditions, miRNAs shown in Table 3 were chosen for the comparative analyses between sedentary and trained subjects. The expression profile of these selected miRNAs are shown in Figure 5. The expression profiles of all the other miRNAs are shown in Figure S1.

**Table 3:** List of the selected miRNAs associated with skeletal muscle and bone with known functions in human.

miRNA	Target	Biological Processes	Ref.
hsa-let-7a-5p	n.d.	Muscle atrophy	^18^
hsa-miR-1	HDAC4, CX43, MyoD, Pax7	Skeletal muscle development and differentiation	^31, 32^
	n.d.	Adaptive response to PA	^20– 22^
hsa-miR-101-3p	n.d.	Muscle atrophy	^18^
	EZH2	Promotion of hMSCs differentiation	^44^
hsa-miR-103a-3p	n.d.	Myogenesis	^17^
	RUNX2	Inhibition of osteoblast differentiation	^45^
hsa-miR-107	n.d.	Myogenesis	^17^
hsa-miR-125b-5p	Osx	Inhibition of hMSCs proliferation and differentiation	^46^
	BMPR1b	Inhibition of hMSCs differentiation	^47^
	n.d.	Bone mineral density (Osteoporosis)	^48^
hsa-miR-140-5p	BMP-2	Inhibition of the osteogenic lineage commitment in undifferentiated hMSCs	^49^
hsa-miR-142-3p	PKCα	Reduction of osteoclast viability	^54^
	APC	Promotion of osteoblast differentiation trough WNT signaling	^50^
hsa-miR-148b-3p	n.d.	Muscle atrophy	^18^
hsa-miR-181a-5p	n.d.	Adaptive response to PA	^22^
hsa-miR-28-3p	n.d.	Myogenesis	^17^
hsa-miR-199a-5p	FDZ, JAG1, WNT2	Maintenance of muscle satellite cells and differentiation of satellite cells into myofibers	^34^
	LIF	Promotion of hMSCs differentiation	^51^
hsa-miR-29a	DKK1, Kremen2, sFRP2	Promotion of osteoblasts differentiation	^52^
	n.d.	Adaptive response to PA	^21^
hsa-miR-29 b/c	YY1	Promotion of skeletal muscle differentiation	^33^
	TRAcP, MMP9, c-FOS, NAFTc-1	Inhibition of osteoclast differentiation	^55^
	n.d.	Adaptive response to PA	^22^
hsa-miR-324-5p	n.d.	Myogenesis	^17^
hsa-miR-331-3p	n.d.	Myogenesis	^17^
hsa-miR-335-5p	RUNX2	Inhibition of hMSCs proliferation, migration and differentiation	^53^
hsa-miR-374a-5p	n.d.	Myogenesis	^17^
hsa-miR-378a-5p	n.d.	Adaptive response to PA	^21^
hsa-miR-424-5p	PolR1A, RRN3, UBTF	Muscle wasting	^19^
hsa-miR-502-3p	n.d.	Myogenesis	^17^
hsa-miR-660-5p	n.d.	Myogenesis	^17^

n.d.: not determined.

Among miRNAs specifically expressed in skeletal muscle (hsa-miR-1, hsa-miR-133a, hsa-miR-133b, hsa-miR-206, hsa-miR-208a, hsa-miR-208b, hsa-miR-486, and hsa-miR-499), commonly called myo-miRNAs, only miR-1 was significantly up-regulated in plasma of trained subjects. hsa-miR-29b-3p, hsa-miR-29c-3p, and other miRNAs expressed in human myoblasts and myotubes and involved in myogenic differentiation^17^ were significantly up-regulated (hsa-miR-28-3p, hsa-miR-29b-3p, hsa-miR-29c-3p, hsa-miR-324-5p, hsa-miR-374a-5p, hsa-miR-502-3p, and hsa-miR-660-5p) or down-regulated (hsa-miR-103a-3p, hsa-miR-107, hsa-miR-199a-5p, and hsa-miR-331-3p) in plasma samples of trained subjects. miRNAs involved in skeletal muscle atrophy and wasting^18,19^ were down-regulated in plasma of trained subject (hsa-let-7a-5p, hsa-miR-101-3p and hsa-miR-424-5p) while hsa-miR-148b-3p was up-regulated. It is known that different types of PA modulate the expression of miR-181a, miR-29a and miR-378, as well as of miR-1 and miR-29b^20–22^ in skeletal muscle. Our results showed that these miRNAs were down-regulated in plasma of trained subjects.

Additionally, several miRNAs involved in osteoblast and osteoclast differentiation were modulated by the trained status. Both promotors and inhibitors of osteoblast differentiation were down-regulated in trained subjects (hsa-miR-101-3p, hsa-miR-103a-3p, hsa-miR-125b-5p, hsa-miR-140-5p, hsa-miR-142-3p, hsa-miR-199a-5p, and hsa-miR-29a-3p) while hsa-miR-335-5p, that negatively regulates osteoblastogenesis, was up-regulated. Among the miRNAs involved in osteoclast differentiation, hsa-miR-29b-3p, that inhibits osteoclastogenesis, was considerably up-regulated; whereas hsa-miR-142-3p, principally involved in osteoclast survival, was down-regulated in trained subjects compared to sedentary.

## Discussion

With this study, we investigated the effect of different normalization strategies on the quantification of a panel of plasma miRNAs. Four main miRNA normalization methods, i.e., global mean, mean-endo, single miRNAs, and mean-spike-in, were assessed and compared to each other in order to identify the best strategy that gives the most reliable results. These methods were chosen based on their wide use in normalizing RT-qPCR data of circulating miRNAs, according to literature^5^. In order to give a physiological contextualization to the post-analytical normalization-dependent variability, we compared two cohorts of subjects: sedentary vs. highly-trained subjects. This choice was driven by the fact that training (i.e., chronic PA), and hence a physically active status, is associated with definitively modified circulating levels of several parameters and, among these, of miRNAs^15^.

Several studies have used endogenous miRNAs for normalization purposes, but the choice was dictated by different reasons (e.g., literature search, software-based identification, arbitrary choice). Resnick *et al*. applied a hsa-miR-142-3p-based normalization to quantify circulating miRNAs in serum of ovarian cancer patients^23^, while in another cohort of cancer patients Hu *et al*. identified hsa-miR-1228 as a reference gene^24^. Using NormFinder and BestKeeper algorithm, Danese *et al*. identified the combination of hsa-miR-1228 and hsa-miR-520d as the most suitable reference to normalize miRNAs from plasma, exosome, and tissue obtained from colorectal cancer patients^25^. Mestdagh *et al*. compared different normalization strategies (i.e., mean of all the expressed miRNAs, stable small RNA controls [RNU24, RNU44, RNU58A, and RNU6B] and miRNA/smallRNA controls resembling the mean expression value) and demonstrated that the mean of all the expressed miRNAs was the most appropriate strategy for large datasets^9^. Similarly, among the studies that used exogenous oligonucleotides as normalizers, Mitchell *et al*. used the average value of cel-39, cel-54 and cel-238 for normalizing serum/plasma miRNAs of prostate cancer patients. McDonald *et al*., in a methodological study on the effect of pre-analytical and analytical variable on circulating miRNA quantification, showed that normalization on cel-39, mean of cel-39 and cel-54, and mean of cel-39, cel-54, and cel-238 reduce the inter-assay variability of miRNA expression more than the endogenous miR-16^26^. In a previous study, we used hsa-miR-425-5p and hsa-miR-484 as reference genes for studying the effect of a PA protocol on fracture risk-associated miRNA^27^.

In this study, a panel of 179 circulating miRNAs, comprising the most expressed and clinically relevant miRNAs^28^ was assayed. The analyses performed on such large panel of miRNAs, without any *a priori* analysis of reference genes to use as normalizers of RT-qPCR data, led us to use NormFinder algorithm^10^, provided by GenEX software, in order to define the potentially best reference genes. Among all the analysed miRNAs, NormFinder selected 33 miRNAs as potential reference genes (Table S1). Considering all the endogenous miRNAs-based normalization strategies, besides the global mean (i.e., the averaged Cq value of the whole data-set) and the mean-endo (i.e., the averaged Cq value of the most stable miRNAs), also normalization on single miRNAs has been evaluated. About this latter approach, the most stable miRNAs in our analysis were chosen by considering an Acc.SD threshold of 50%, which led to the selection of the first eight miRNAs of the list. As a term of comparison, also the last three less stable miRNAs, ranked by NormFinder, were selected. Also, exogenous oligonucleotides, UniSp2, UniSp4, UniSp6 and cel-39, were assessed as normalizers for RT-qPCR data. Our results demonstrated that the reliability of the analytical output, indicated by a lower CV, was improved when using the single miRNA hsa-miR-320d, while an increased variability between the experimental replicates was obtained following normalization with the less stable endogenous miRNAs and exogenous oligonucleotides.

The effects of different normalization strategies on the analytical output obtained from circulating miRNAs determination was assayed in a “real-life” situation as the comparison between two different, in some ways opposite, physiological models: sedentary and highly-trained subjects. These subjects were selected based on their physical active status according to the guidelines of the American College of Sports Medicine (ACSM). Sedentary are defined as those subjects performing less than a total of 150 min per week (or less than 5 sessions a week, 30 min per session) of moderate intensity PA^29^. The trained group, instead, was composed of subjects accustomed to high volumes of vigorous-to-high intensity PA being them habitual ultra-trail runners. Importantly, these subjects were sampled the day before an ultra-trail running in order to limit the effect of other variables; indeed, the training regimen in view of a competition is strikingly defined and, moreover, it expects a tapering period, the week before the competition, during which the training volume is halved. For the same reason, the sedentary subjects were asked to comply with specific behaviors and to avoid PA and pro-inflammatory activities.

Our results revealed that normalizing on either global mean, mean-endo, hsa-miR-21a-5p, and hsa-miR-320d gave very similar expression profiles for all the target miRNAs. However, by virtue of the lower CV values, only the hsa-miR-320d-based normalization allowed to reach the statistical significance in the comparisons. Indeed, as shown by the CV% analyses, hsa-miR-320d-based normalization reduced the technical variability among the experimental replicates more effectively than other normalization strategies. Therefore, in this specific experimental setting, has-miR-320d was the most appropriate reference gene. On the other hand, the analytical output obtained by normalizing on exogenous oligonucleotides showed different, or even inverted expression profiles, compared to those obtained with the other normalization methods, indicating that this normalization strategy might lead to misinterpretation of the results (Fig. 3).

It is clear that different normalization methods profoundly affect the final result and, hence, they strongly influence the experimental outcome. This is of great clinical relevance because different normalization strategies can give completely different analytical outputs and this can eventually lead to an erroneous diagnosis^12^. Indeed, as recently published, normalization on either cel-miR-39, has-miR-16, or their combination gave very different results of miRNA expression^30^.

Beside the acute effect of exercise, several studies investigated the chronic PA-dependent modulation of circulating miRNAs, demonstrating that different trainings affect their expression and, hence, their circulating levels, although in a different manner^13^. In our study, among the 179 most relevant circulating miRNAs, 40 miRNAs were ≥5-fold up-regulated and 17 were ≥5-fold down-regulated in trained subjects compared to sedentary subjects (Table 2). Of these, 23 miRNAs have been previously associated to skeletal muscle and bone physiology in human (Table 3). From this study emerged that the influence of the training status on skeletal muscle physiology takes place through the up-regulation of hsa-miR-1^31,32^, hsa-miR-148b-3p^18^, hsa-miR-28-3p^17^, hsa-miR-29b-3p^33^, hsa-miR-29c-3p^33^, hsa-miR-324-5p^17^, hsa-miR-374a-5p^17^, hsa-miR-502-3p^17^, and hsa-miR-660-5p^17^ and down-regulation of hsa-let-7a-5p^18^, hsa-miR-101-3p^18^, hsa-miR-103a-3p^17^, hsa-miR-107^17^, hsa-miR-181a-5p^22^, hsa-miR-199a-5p^34^, hsa-miR-29a-3p^21^, hsa-miR-331-3p^17^, hsa-miR-378a-3p^21^, and hsa-miR-424-5p^19^. miR-1 has been found modulated by exercise both in skeletal muscles and in circulation: its expression in skeletal muscle was induced by an acute bout of endurance training, while it was downregulated after 12 weeks of endurance training^20^. On the contrary, other studies revealed the up-regulation of circulating miR-1 after a marathon^35,36^. Davidsen *et al*., failed to detect any changes in the expression of miR-1 and other myo-miRNAs in skeletal muscle following resistance training (12 weeks, 5 days a week) in 56 young untrained men^21^. miR-29b expression is induced by short-term endurance training in skeletal muscle tissue^22^ but its plasma levels were down-regulated immediately and up to five days after a marathon and after 12-week endurance training^37,38^. miR-181 was up-regulated in skeletal muscle biopsies from healthy untrained male after an acute bout of endurance exercise^22^, while miR-29a and miR-378a were downregulated by resistance PA in low responders^21^. In response to endurance training (12 weeks, 3-to-5 days a week), miR-103 and miR-107 were up-regulated in plasma of young healthy men, while miR-148b was down-regulated^38^.

However, many of the miRNAs we found differentially modulated between our cohorts were functionally characterized only in experimental models (e.g., hsa-miR-125b, miR-155, and hsa-miR-30 family members)^39–41^. In a study of De Gonzalo-Calvo investigating the effect of exercise on systemic inflammation, miR-125b-5p, miR-29a-3p, miR-424-5p and miR-132-3p were up-regulated in serum samples of active middle-aged males immediately after a marathon race^42^. These miRNAs, however, were down-regulated in our experimental setting, except for miR-132-3p that was up-regulated; these results support the hypothesis of training-induced adaptation, beside the inflammatory milieu and the effect of acute exercise.

In our analysis, we also highlight the effects on bone function. The knowledge about the PA-associated changes in bone-derived miRNAs are very poor, while they are mostly related to bone disorders and osteoporosis^43^. Here, we have found that a trained status is associated with a defined profile of circulating miRNAs involved in the regulation of both osteoblastogenesis (hsa-miR-101-3p^44^, hsa-miR-103a-3p^45^, hsa-miR-125b-5p^46–48^, hsa-miR-140-5p^49^, hsa-miR-142-3p^50^, hsa-miR-199a-5p^51^, miR-29a-3p^52^, and hsa-miR-335-5p^53^) and osteoclastogenesis (hsa-miR-142-3p^54^ and hsa-miR-29b-3p^55^). Noteworthy, hsa-miR-125b-5p and hsa-miR-122-5p, which were associated with fracture risk^48^, were down-regulated in our endurance trained cohort as well as in a cohort of young untrained males after an 8-week sprint training protocol^27^.

Other miRNAs associated to bone, whose function were not previously characterized in human (i.e., hsa-miR-155-5p^56,57^, hsa-miR-181a-5p^58^, hsa-miR-30c-5p^59^, hsa-miR-378a-3p^60,61^, and hsa-miR-93-3p^62^) were modulated in trained subjects. Taken together these results suggest that chronic PA (i.e., training) alters the expression profile of several miRNAs which mark the skeletal muscle and bone adaptation to such a stimulus.

Besides the obtained results, this study suffers of some limitations. First, the experimental setting, specifically drawn to investigate the effect of the normalization strategy, does not allow to determine how current PA differently affects the circulating miRNAs profile in sedentary and trained subjects. Indeed, the analysis of circulating miRNAs on the same group of individuals before and after a single bout of acute exercise would have better highlighted the physiological response to exercise. Noteworthy, the choice of these two cohorts derives from a major recurrent concern in laboratory medicine: the lack of any reference ranges specific for the athlete populations^15^. Secondly, the analysis of skeletal muscle miRNAs and the definition of their relationship with the circulating counterpart would give additional information about the physiological response to chronic training. However, the issues around the post-analytical data processing equally affect tissue and circulating miRNAs; furthermore, tissue miRNAs analysis is as challenging as circulating miRNAs determination^12^.

The importance of studying the relative differences in circulating miRNAs between sedentary and trained subjects resides in the need to understand how much, and in which way, PA influences the blood levels of these molecules that are next to be introduced in different clinical settings^13^. Indeed, PA is an important pre-analytical variable affecting several biochemical and hematological parameters, throughout different mechanisms, e.g., hemodynamic changes (i.e., hemodilution/hemoconcentration), myocyte damage and release of their intracellular content, direct modification of metabolic activity of cells, endocrine function, and inflammatory phenotype^63^. Nonetheless, PA is considered one of the best and most effective strategy to improve the overall health status, often “prescribed” in association to pharmacological treatment^64^. Hence, the knowledge of the exercise-associated modifications of circulating miRNA profile could be useful in monitoring the effectiveness of an intervention. Exercise is responsible for the modification of the circulating levels of a huge panel of miRNAs and, especially, those associated with muscle fibres (skeletal and cardiac) damage/membrane leakage, contraction machinery regulation, and calcium signalling^38,65^, oxygen consumption, hypoxia, and angiogenesis^38,66^, inflammation and aging^67^, bone metabolism^27,68^. Consequently, studies on circulating miRNAs need a strict definition of the pre-analytical variables in order to minimize the number of confounding factors that affect the final analytical output^69^.

Besides the pre-analytical (e.g., choice of sample matrix, samples handling and storage, lifestyle factors)^15^ and analytical issues (e.g., low concordance between different platforms)^69^, the post-analytical concerns, and mainly the normalization strategy, strongly limits the clinical implementation of miRNA. Hence, as evidenced also in our study, the issue about a correct and universally accepted method of quantification is still unsolved.

## Materials and Methods

### Sample collection

Twenty-four male subjects were recruited and grouped based on their physical activity profile. A first group (sedentary: <30 min, 5 days/week or 150 min/week of moderate intensity activity, according to ACSM guidelines)^29^, was composed of 10 males (age range: 25–40 years), used as control. Individuals were considered eligible if healthy, not suffering for any chronic disease, not affected by any acute seasonal disease within the month preceding the recruitment, not taking any chronic or current medication, with a normal body mass index (BMI, between 20 and 25 kg/m^2^), and non-smoker. Participants were asked to abstain from alcohol consumption and from any PA in the 48 h before the blood sampling, as well as to avoid any pro-inflammatory activity (shaving, waxing, etc.) in the same timeframe, and to keep a regular diet. The second group (trained) was composed of 14 volunteer male mountain ultra-trail non-professional athletes (age range: 25–40 years; BMI between 20 and 25 kg/m^2^) accustomed to high volumes of long-lasting endurance PA performed at vigorous-to-high intensities according to the guidelines from the ACSM^29^. Specifically, these subjects came from an 8-week preparation period to the race during which they trained 4-to-5 times per week with exercise series performed at intensities ranging between 70% and 90% of the maximal heart rate (HR_max_, excluding the warm up). Typically, the exercise sessions during the week were: (1) resistance exercise (80% HR_max_), (2) plain running (70% HR_max_) + uphill repeated sprints + light running, (3) uphill and/or downhill running (70% HR_max_), (4) plain running (80% HR_max_) + uphill and/or downhill running (90% HR_max_), (5) plain running (80% HR_max_) + uphill and/or downhill running (90% HR_max_) The total amount of hours per week increased from 8 h to 10h30min during this period. The week before the competition, i.e., the ninth week, the training volume was reduced of 50%, a recovery strategy commonly named tapering^70^. These subjects were sampled at the end of the pre-competition tapering period, the day before an ultra-trail race. Venous blood of each individuals was collected in K2EDTA tubes (BD Vacutainer^®^, Becton Dickinson, Franklin Lakes, NJ, USA) and plasma was obtained following centrifugation at standard conditions of 1300 *g* for 10 min at room temperature (22 °C). Plasma aliquots were immediately frozen at −80 °C until assayed. In accordance with the Declaration of Helsinki, all subjects were informed about the procedures, benefits, and possible adverse events associated with the protocol. All subjects gave their written informed consent for study participation. The protocol was approved by the Comitato Etico Ospedale San Raffaele, Milano, Italia (SportMarker, ClinicalTrials.gov Registration Number NCT03386981, registered on 4^th^ January 2018).

### miRNA profiling

miRNA-enriched total RNA was extracted from plasma with miRCURY™ RNA Isolation Kit (Exiqon A/S, Vedbaek, Denmark). Extraction efficiency was checked through three synthetic oligonucleotides (spike-ins: UniSp2, UniSp4, UniSp5) added at recommended concentrations. Reverse transcription was performed with miRCURY LNA™ Universal RT miRNA PCR, using UniSp6 and cel-39 as internal controls. RT-qPCR reaction was carried out using serum/plasma miRCURY LNA™ miRNA focus panel (Exiqon), containing the primer set of 179 microRNA, the most expressed in circulation^28^. RT-qPCR was carried out on a StepOne Plus instrument (Applied Biosystem, Foster City, CA, USA), using ExiLENT SYBR Green Master Mix. Polymerase activation for 10 min at 95 °C was followed by 40 amplification cycles of 10 s each at 95 °C, 60 s at 60 °C, and by the melting curve. Results, reported as Cq value, were analyzed by the Exiqon GenEx software ver6. Outlier values obtained from abnormal amplification were removed from the input files of GenEx analyses. The Cq values obtained from the different panels were adjusted by an Inter Plate Calibrator (IPC). Each panel contains three IPC with specific primer pairs and DNA template; the Cq value of each IPC was used to calibrate qPCR plate runs of different experiments. Only those miRNAs with a Cq <37 were considered for the analysis. The relative expression of each miRNA was calculated by the 2^−ΔΔCq^ method and normalized on global mean (mean of the Cq of all expressed miRNAs), mean-endo (mean of the Cq of the most stable miRNAs), single endogenous miRNA, on mean-spike-in (mean of the Cq of the exogenous oligonucleotides), and Cq of single exogenous oligonucleotides. Hemolysis was checked by the hsa-miR-23-a-to- hsa-miR-451 ΔCq ratio (positive if >7).

### Identification of stable miRNAs

The analysis of the expression stability was performed on both endogenous miRNAs and exogenous oligonucleotides (i.e., the spike-in added during microRNA extraction and reverse-transcription) by using the NormFinder algorithm^10^ supplied by the GenEx software. Norm Finder is an algorithm that ranks candidate genes based on their expression stability and identifies the most stable to be used as suitable reference genes for normalization. The most stably expressed genes are characterized by a lower arbitrary stability value calculated by the algorithm itself. Furthermore, NormFinder calculates the accumulated standard deviation (Acc.SD). When using larger number of genes, the comparison of the arbitrary stability value of each gene gives a minimum in the Acc.SD plot, which indicates the number of genes to be used as reference genes for normalization.

### Statistical analysis

Statistical analysis was performed with Prism^®^ v6.01 (GraphPad Software). All data were compared throughout unpaired t-test with Welch’s correction. The differences were considered significant when p value < 0.05 (*p < 0.05, **p < 0.01, ***p < 0.001).

## Supplementary information

Figure S1

Table S1

## Data Availability

The datasets generated during and/or analyzed during the current study are available from the corresponding author on reasonable request.
