# Supplementary material for: Normalization strategies differently affect circulating miRNA profile associated with the training status
Source: Sci Rep. 2019 Feb 7;9:1584. doi: 10.1038/s41598-019-38505-x (PMC6367481; doi:10.1038/s41598-019-38505-x)
Supplement: Supplementary file 1 — Figure S1 [file 41598_2019_38505_MOESM1_ESM.pdf]

Normalization strategies differently affect circulating miRNA profile associated with the training status

Martina Faraldi[1] *, Marta Gomarasca[1], Veronica Sansoni[1], Silvia Perego[1], Giuseppe Banfi[1,2], Giovanni Lombardi[1]

1. Laboratory of Experimental Biochemistry & Molecular Biology, IRCCS Istituto Ortopedico Galeazzi, Milano, Italia
2. Vita-Salute San Raffaele University, Milano, Italia

Figure S1. Expression profile of miRNAs ≥ ± 5-fold up- or down-regulated in plasma of sedentary and trained subjects. Expression profile of miRNAs associated to skeletal muscle and bone, which function was defined in experimental models rather than in humans. Statistical analysis was performed with Prism v6.01 (GraphPad Software). All data are expressed as mean ±SEM and data were compared throughout t-test with Welch's correction. The differences were considered significant when p value < 0.05 (*$p<0.05$, **$p<0.01$, ***$p<0.001$).

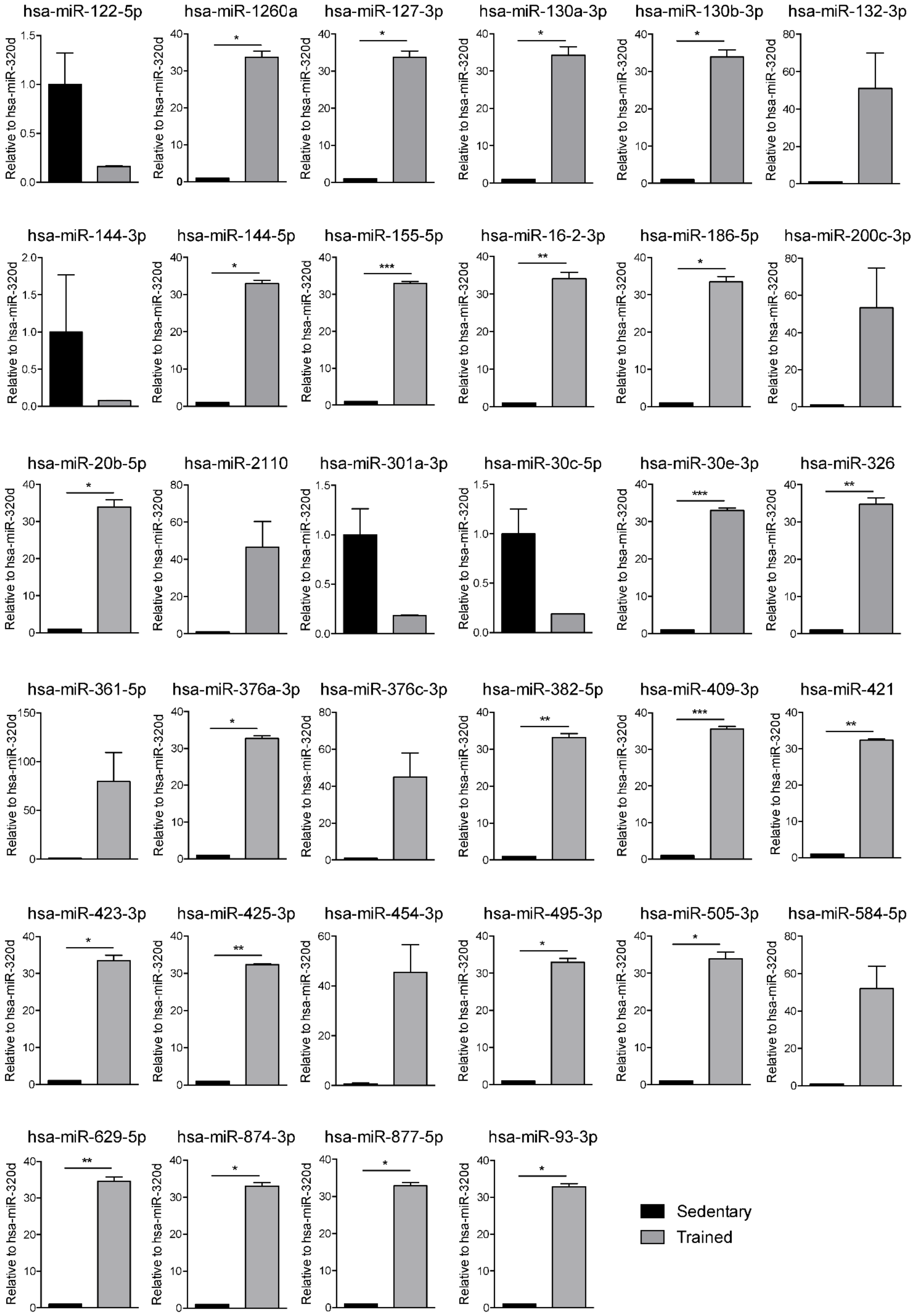
hsa-miR-122-5p
hsa-miR-1260a
hsa-miR-127-3p
hsa-miR-130a-3p
hsa-miR-130b-3p
hsa-miR-132-3p
hsa-miR-144-3p
hsa-miR-144-5p
hsa-miR-155-5p
hsa-miR-16-2-3p
hsa-miR-186-5p
hsa-miR-200c-3p
hsa-miR-20b-5p
hsa-miR-2110
hsa-miR-301a-3p
hsa-miR-30c-5p
hsa-miR-30e-3p
hsa-miR-326
hsa-miR-361-5p
hsa-miR-376a-3p
hsa-miR-376c-3p
hsa-miR-382-5p
hsa-miR-409-3p
hsa-miR-421
hsa-miR-423-3p
hsa-miR-425-3p
hsa-miR-454-3p
hsa-miR-495-3p
hsa-miR-505-3p
hsa-miR-584-5p
hsa-miR-629-5p
hsa-miR-874-3p
hsa-miR-877-5p
hsa-miR-93-3p
Relative to hsa-miR-320d
Sedentary
Trained
