# Supplementary material for: Normalization strategies differently affect circulating miRNA profile associated with the training status
Source: Sci Rep. 2019 Feb 7;9:1584. doi: 10.1038/s41598-019-38505-x (PMC6367481; doi:10.1038/s41598-019-38505-x)
Supplement: Supplementary file 2 — Table S1 [file 41598_2019_38505_MOESM2_ESM.pdf]

Normalization strategies differently affect circulating miRNA profile associated with the training status

Martina Faraldi[1] *, Marta Gomarasca[1], Veronica Sansoni[1], Silvia Perego[1], Giuseppe Banfi[1,2], Giovanni Lombardi[1]

1. Laboratory of Experimental Biochemistry & Molecular Biology, IRCCS Istituto Ortopedico Galeazzi, Milano, Italia
2. Vita-Salute San Raffaele University, Milano, Italia

Supplementary Table 1. List of the most stable miRNAs ranked by NormFinder.

NAME	*ACCESSION NUMBER*	*GENOME CONTEXT*	*CLUSTER*
**hsa-miR-21-5p**	MIMAT0000076	chr17	-
**hsa-miR-126-3p**	MIMAT0000445	chr9	-
**hsa-miR-125a-5p**	MIMAT0000443	chr19	hsa-miR-99b, hsa-let-7e, hsa-miR-125a
**hsa-miR-590-5p**	MIMAT0003258	chr7	-
**hsa-miR-26b-5p**	MIMAT0000083	chr2	-
**hsa-miR-320d**	MI0008190	chr13	-
**hsa-miR-23a-3p**	MIMAT0000078	chr19	hsa-miR-23a, hsa-miR-27a, hsa-miR-24-2
**hsa-miR-146a-5p**	MIMAT0000449	chr5	-
**hsa-miR-223-3p**	MIMAT0000280	chrX	-
**hsa-miR-93-5p**	MIMAT0004509	chr7	hsa-miR-106b, hsa-miR-93, hsa-miR-25
**hsa-miR-22-3p**	MIMAT0000077	chr17	-
**hsa-miR-30e-5p**	MIMAT0000692	chr1	hsa-miR-30e, hsa-miR-30c-1
**hsa-let-7g-5p**	MIMAT0000414	chr3	-
**hsa-miR-18a-5p**	MIMAT0000072	chr13	hsa-miR-17, hsa-miR-18a, hsa-miR-19a, hsa-miR-20a, hsa-miR-19b-1, hsa-miR-92a-1
**hsa-miR-142-5p**	MIMAT0000433	chr17	hsa-miR-4736, hsa-miR-142
**hsa-miR-374b-5p**	MIMAT0004955	chrX	hsa-miR-374c, hsa-miR-374b, hsa-miR-421
hsa-miR-19a-3p	MIMAT0000073	chr13	hsa-miR-17, hsa-miR-18a, hsa-miR-19a, hsa-miR-20a, hsa-miR-19b-1, hsa-miR-92a-1
**hsa-miR-199a-3p**	MIMAT0000232	chr19<br>chr1	-<br>hsa-miR-199a-2, hsa-miR-3120, hsa-miR-214
hsa-miR-25-3p	MIMAT0000081	chr7	hsa-miR-106b, hsa-miR-93, hsa-miR-25
**hsa-miR-150-5p**	MIMAT0000451	chr19	-
**hsa-miR-145-5p**	MIMAT0000437	chr5	hsa-miR-143, hsa-miR-145
hsa-miR-24-3p	MIMAT0000080	chr9<br>chr19	hsa-miR-23b, hsa-miR-27b, hsa-miR-3074, hsa-miR-24-1<br>hsa-miR-23a, hsa-miR-27a, hsa-miR-24-2
**hsa-miR-106a-5p**	MIMAT0000103	chrX	hsa-miR-106a, hsa-miR-18b, hsa-miR-20b, hsa-miR-19b-2, hsa-miR-92a-2, hsa-miR-363
**hsa-miR-185-5p**	MIMAT0000455	chr22	-

**hsa-miR-197-3p**	MIMAT0000227	chr1	-
**hsa-miR-766-3p**	MIMAT0003888	chrX	-
**hsa-miR-192-5p**	MIMAT0000222	chr11	hsa-miR-6750, hsa-miR-194-2, hsa-miR-192
**hsa-miR-425-5p**	MIMAT0003393	chr3	hsa-miR-191, hsa-miR-425
**hsa-miR-151a-5p**	MIMAT0004697	chr8	-
**hsa-miR-30d-5p**	MIMAT0000245	chr8	hsa-miR-30d, hsa-miR-30b
**hsa-miR-222-3p**	MIMAT0000279	chrX	hsa-miR-222, hsa-miR-221
**hsa-miR-27b-3p**	MIMAT0000419	chr9	hsa-miR-23b, hsa-miR-27b, hsa-miR-3074, hsa-miR-24-1
hsa-miR-106b-5p	MIMAT0000680	chr7	hsa-miR-106b, hsa-miR-93, hsa-miR-25
**hsa-let-7b-5p**	MIMAT0000063	chr22	hsa-let-7a-3, hsa-mir-4763, hsa-let-7b
hsa-miR-19b-3p	MIMAT0000074	chr13<br>chrX	hsa-miR-17, hsa-miR-18a, hsa-miR-19a, hsa-miR-20a, hsa-miR-19b-1, hsa-miR-92a-1<br>hsa-miR106a, hsa-miR-18b, hsa-miR-20b, hsa-miR19b-2, hsa-miR-92a-2, hsa-miR363
**hsa-miR-484**	MI0002468	chr16	-
hsa-miR-191-5p	MIMAT0000440	chr3	hsa-miR-191, hsa-miR-425
hsa-miR-20a-5p	MIMAT0000075	chr13	hsa-miR-17, hsa-miR-18a, hsa-miR-19a, hsa-miR-20a, hsa-miR-19b-1, hsa-miR-92a-1
**hsa-miR-423-5p**	MIMAT0004748	chr17	hsa-miR-432, hsa-miR-3184
**hsa-let-7i-5p**	MIMAT0000415	chr12	-
hsa-miR-30b-5p	MIMAT0000420	chr8	hsa-miR-30d, hsa-miR-30b

The table shows the 41 most stable gene ranked by NormFinder. For each miRNA accession number, genome context, and cluster belongings are indicated. In bold are pointed out the first stable miRNAs of each cluster, namely the 33 miRNAs, considered in normalization
